# The Role and Efficacy of Vitamin C in Sepsis: A Systematic Review and Meta-Analysis

**DOI:** 10.3390/arm90040038

**Published:** 2022-07-28

**Authors:** Marwah Muhammad, Ahmad Jahangir, Ali Kassem, Saud Bin Abdul Sattar, Abdullah Jahangir, Syeda Sahra, Muhammad Rafay Khan Niazi, Ahmad Mustafa, Zeeshan Zia, Fasih Sami Siddiqui, Waleed Sadiq, Danil Mishiyev, Aleena Sammar, Loai Dahabra, Aazib Irshad, Dany Elsayegh, Michel Chalhoub

**Affiliations:** 1 Sahiwal Medical College, Sahiwal 57000, Pakistan; mmarwah02@gmail.com; 2 Mayo Hospital, Lahore 54000, Pakistan; ahmadjahangir786@gmail.com; 3 Staten Island University Hospital, Northwell Health, Staten Island, New York, NY 10305, USA; akassem@northwell.edu (A.K.); ssattar1@northwell.edu (S.B.A.S.); abdullahjahangir6103@gmail.com (A.J.); mniazi@northwell.edu (M.R.K.N.); amustafa@northwell.edu (A.M.); zzia@northwell.edu (Z.Z.); fsiddiqui9@northwell.edu (F.S.S.); wsadiq@northwell.edu (W.S.); dmishiyev@northwell.edu (D.M.); ldahabra@northwell.edu (L.D.); delsayegh@northwell.edu (D.E.); mchalhoub1@northwell.edu (M.C.); 4 Pakistan Institute of Medical Sciences, Islamabad 44000, Pakistan; aleenasammar@gmail.com; 5 Jinnah Hospital, Lahore 54000, Pakistan; aazib05@gmail.com

**Keywords:** sepsis, septic shock, ICU length of stay, hospital length of stay, vitamin C, ascorbic acid

## Abstract

**Highlights:**

- Sepsis is a significant global healthcare burden and the leading cause of in-patient mortality.
- Our analysis showed that vitamin C adjunct therapy was linked to a significant decrease in mortality, duration of vasopressor used, and SOFA scores in septic patients.
- There is a further need for a large, randomized control trial to define a clearer role of vitamin C in the care of septic patients.

**Abstract:**

Clinical rationale for study: Despite advancements in critical care, the mortality rate of sepsis remains high, with an overall poor prognosis. There is a complex pathophysiology of a lethal cascade of cytokines and inflammatory proteins underlying sepsis. The use of vitamin C can theoretically suppress the inflammatory cascade but remains a questionable practice due to a lack of conclusive evidence. Aims of the study: To appraise the therapeutic role of vitamin C in sepsis. Materials and methods: A systematic review was conducted on PubMed, Embase, and the Central Cochrane Registry. The study included randomized clinical trials (RCTs) with vitamin C as an intervention arm in the septic patient population. For continuous variables, the difference in means (MD) and for discrete variables, the odds ratio (OR) was used. For effect sizes, a confidence interval of 95% was used. A *p*-value of less than 0.05 was used for statistical significance. The analysis was performed using a random-effects model irrespective of heterogeneity. Heterogeneity was evaluated using the I^2^ statistic. Results: 23 studies were included with the total sample size of 2712 patients. In patients treated with vitamin C, there was a statistically significant reduction in the mortality: OR = 0.778 (0.635 to 0.954), *p* = 0.016; the sequential organ failure assessment score (SOFA): MD = −0.749 (−1.115 to −0.383), *p* < 0.001; and the duration of vasopressor requirement: MD = −1.034 days (−1.622 to −0.445), *p* = 0.001. No significant difference was found in the hospital or ICU length of stay. Conclusions and clinical implications: Vitamin C treatment regimens were associated with reduced mortality, SOFA score, and vasopressor requirement compared to the control in sepsis. Given its low cost and minimal adverse effects, we strongly encourage further large, randomized trials to establish vitamin C as a standard of care in sepsis management.

## 1. Introduction

Sepsis is a syndrome characterized by a host’s amplified dysregulated immune response to an underlying infection, leading to multi-organ malfunction. Despite advanced antimicrobials and therapeutics, sepsis has been linked with high mortality and morbidity [1]. Endotoxins such as lipopolysaccharide (LPS component of the cell membranes of Gram-negative bacteria) and other pathogen products can provoke the macrophages to secrete proinflammatory cytokines. This overwhelming immune response from the cytokines, including interleukin-6 (IL-6), interleukin-8 (IL-8), interleukin-1b (IL-1b), macrophage inflammatory protein-1b, and tumor necrosis factor (TNF-a) leads to sequelae of sepsis via capillary leakage and organ failure [2,3].

Globally and nationally, sepsis carries a major burden in morbidity and mortality on the health care system [4,5]. For instance, in 2017, approximately 11 million patients died due to sepsis. Fortunately, the incidence and mortality related to sepsis have decreased. In 1990, approximately 60.2 million sepsis cases were reported compared to 48.9 million cases in 2017. In the United States, sepsis is still the most common cause of death in hospitalized patients. Additionally, sepsis costs the health care system up to 24 billion dollars [6].

Ascorbic acid, more commonly known as vitamin C, is a vital trace element for the human body. Vitamin C is one of the most efficacious water-soluble antioxidants. The human body cannot form vitamin C, an essential nutritional element, and must be supplemented nutritionally. The role of vitamin C in treating multiple diseases has been significant due to its potent antioxidative properties [7]. The low cost of vitamin C, easy availability, and relatively safe side effect profile make it an attractive prospect as an adjunct to sepsis management.

### Clinical Rationale for the Study

Multiple trials have been conducted showing the effectiveness of vitamin C in patients needing intensive care in combination with other drugs or alone. Multiple meta-analyses have reported variable outcomes with conflicting results. We performed this meta-analysis to pool the data from all the previous prospective, randomized control trials to increase the sample size and statistical power to evaluate the role of vitamin C in sepsis.

## 2. Materials and Methods

The study was performed according to the Preferred Reporting Items for Systematic Reviews and Meta-Analyses (PRISMA) guidelines.

### 2.1. Eligibility Criteria

The studies that were included:

1. were RCTs with supraphysiologic doses of vitamin C as an intervention in septic patients with the standard of care as the control arm.
2. had patients aged > 18 years.
3. were available in the English language regardless of date or status of publications.

Studies that did not meet the above criteria were excluded.

### 2.2. Information Sources

The Central Cochrane Registry of clinical trials, Embase, and the PubMed database were used to search relevant articles. A MeSH search with keywords was performed in PubMed, and the PICO search tool was used to search Embase. The MeSH terms included “vitamin C” or “ascorbic” or “ascorbate” and “sepsis” or “septic” or “shock.” All the search terms were exploded without any limitations. The included articles were studied to identify any missing studies. The deadline for publication was set as 31 January 2022.

### 2.3. Trial Selection and Evaluation

Three authors independently and objectively reviewed all studies and excluded unqualifying articles. The risk of bias for the selected studies was calculated using the Cochrane collaborative tool and categorized into low, uncertain, and high.

The quality of evidence was graded using the GRADE system [8]. Each outcome was rated for quality, reducing the level of evidence for risk of bias, inconsistency, indirectness, and imprecision.

### 2.4. Data Extraction

Information was obtained using a pre-specified extraction table. Information was retrieved from the published trials and their tables/appendices, and a second author reviewed the information collected to ensure accuracy. The extracted data included the number of patients, mortality, sequential organ failure assessment score (SOFA) score, hospital length of stay, intensive care unit (ICU) length of stay, and days on pressors.

The primary outcome assessed was short-term mortality, and other outcomes included change in SOFA score, ICU length of stay, duration of vasopressor requirement, and hospital length of stay.

### 2.5. Statistical Analysis

The meta-analysis was performed using the Comprehensive Meta-analysis Software Version 3 (Biostat USA). We calculated the mean difference in continuous variables for treatment effect measurement while the odds ratio was calculated for discrete variables. Where medians and interquartile ranges were reported, they were assessed for skewness and whether the data were similar to a normal distribution, and the mean and standard deviation were imputed [9,10,11]. Standard errors were calculated using a 95% confidence interval, and a *p*-value of 0.05 was used to determine statistical significance. The analysis was performed using a random-effects model for consistency in analysis irrespective of heterogeneity. Heterogeneity was evaluated using the I^2^ statistic; heterogeneity less than 40 was considered low, 40–60 moderate, and above 60 as high [8].

## 3. Results

### 3.1. Literature Search

**Figure 1:**
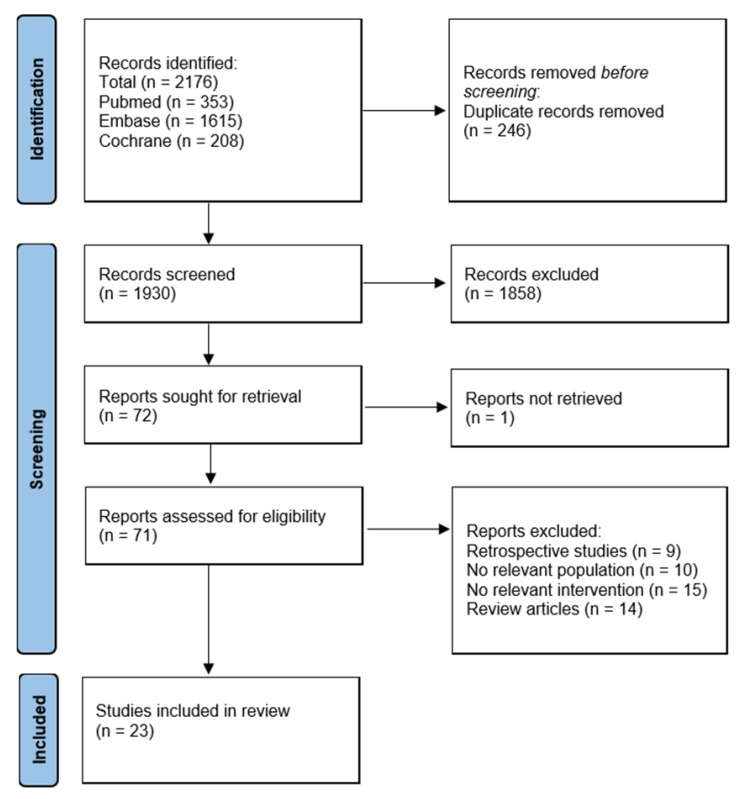
Prisma Diagram.

A total of 2176 articles were identified in the initial search. After the removal of duplicates, 1930 articles were filtered. The first screening excluded 1858 articles. Full texts of 72 were analyzed. Nine articles were excluded due to being retrospective studies; ten did not have a relevant patient population (burn, etc.), fourteen were review articles, fifteen did not have a relevant intervention, and data reports from one study were not retrieved. Twenty-three randomized control trials were included, with a total of 2712 patients (Figure 1). The main characteristics are given in Table 1. 

### 3.2. Risk of Bias

Allocation: Most studies provided details about the methodology of randomization, except one study [32].

Blinding: Most trials were double-blind, except for open-label studies [23,29].

Incomplete data outcomes: 20 studies were considered a low risk for incomplete data, and 3 were deemed an unclear risk [31,32,33].

Selective reporting: 2 studies were deemed an unclear risk for selective reporting [31,33], and one study did not provide reasons for excluding individuals from the analysis and was deemed at a high risk for bias [28].

**Figure 2:**
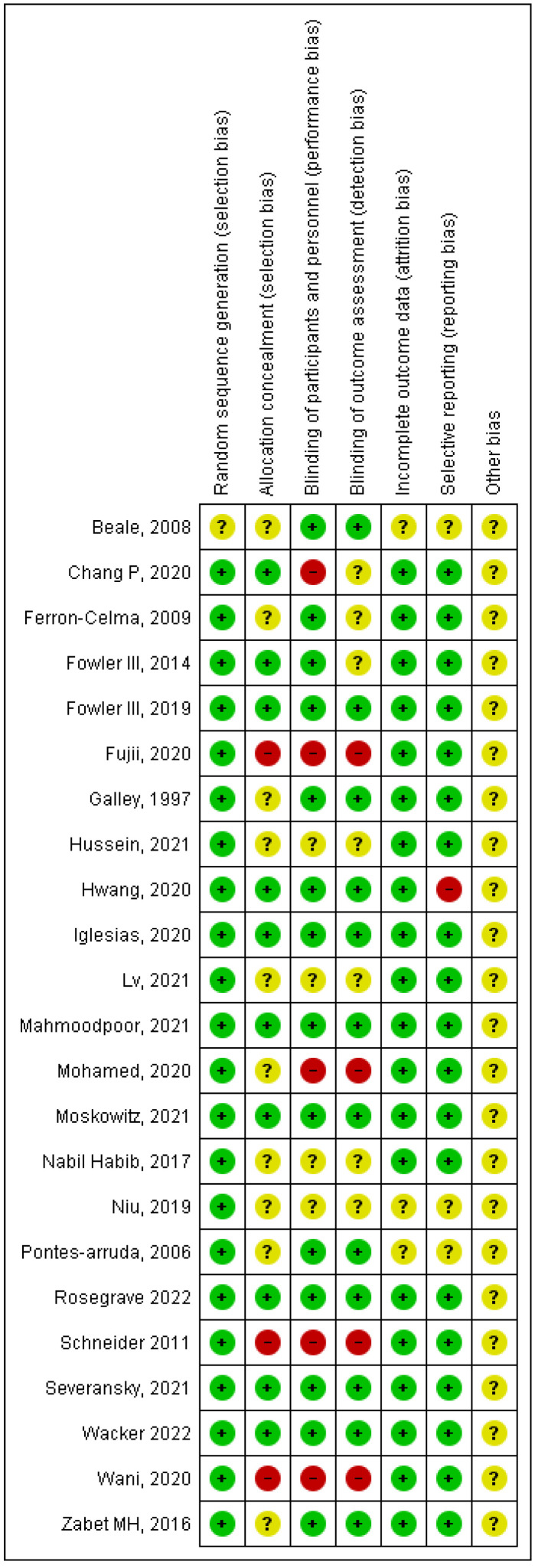
Summary of bias.

**Figure 3:**
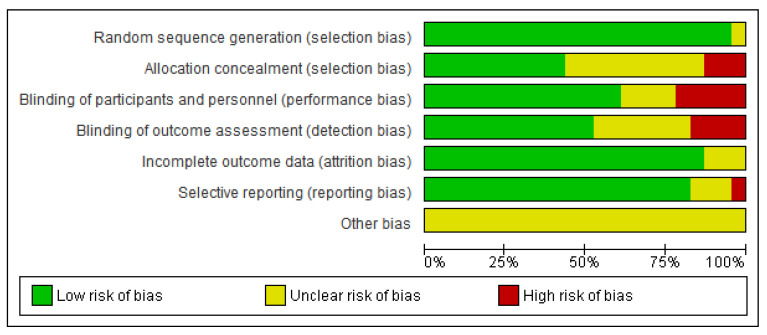
Summary of bias.

The results of the risk of bias are shown in Figure 2 and Figure 3. Overall, most of the information obtained from the studies was deemed to have a low to unclear risk of bias.

### 3.3. Publication Bias

**Figure 4:**
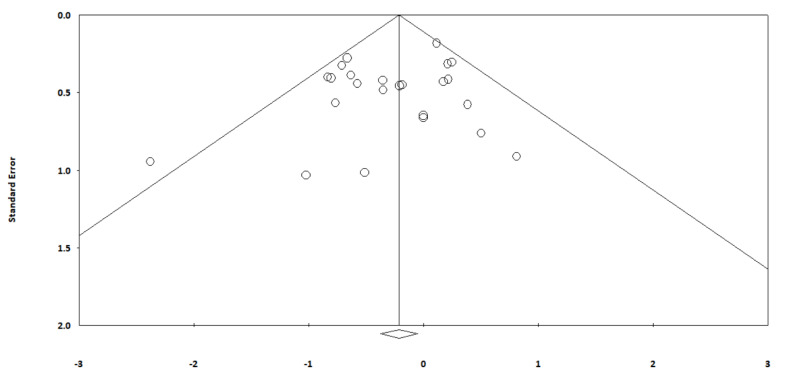
Funnel plot for publication bias (Mortality).

The risk of publication bias was assessed using a funnel plot for mortality. No evidence of significant publication bias was detected (Figure 4).

### 3.4. Results of Quantitative Analysis

#### 3.4.1. Mortality

##### Overall

**Figure 5:**
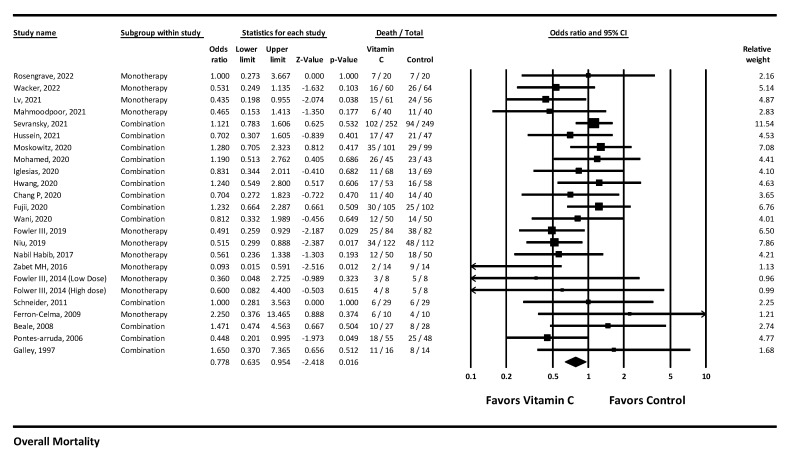
Overall mortality.

A total of 23 studies reported mortality with vitamin-C-containing therapies. Their use was associated with a statistically significant reduction in mortality with OR = 0.778 (0.635 to 0.954), *p* = 0.016, and I^2^ = 25.688 (low heterogeneity, low certainty of evidence) (Figure 5).

##### Intravenous Dosing

Twenty studies reported mortality with intravenous regimens containing vitamin C. Their use was associated with a statistically significant reduction in mortality; OR = 0.780 (628 to 0.968), *p* = 0.024, and I^2^ = 27.690 (low heterogeneity, low certainty of evidence).

##### Enteral Dosing

Three studies reported mortality with enteral regimens containing vitamin C. Their use was not associated with a statistically significant change in mortality; OR = 0.782 (0.367 to 1.665), *p* = 0.524, and I^2^ = 36.416 (low heterogeneity, very low certainty of evidence).

#### 3.4.2. SOFA Score

**Figure 6:**
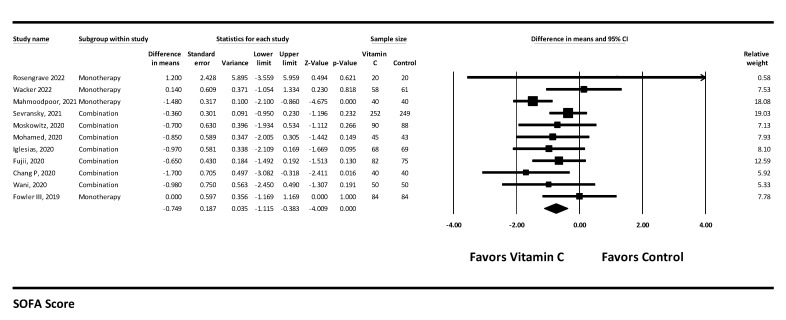
SOFA score.

Eleven studies reported a change in SOFA score with vitamin-C-containing treatment regimens. Their use was associated with a statistically significant reduction in SOFA score with MD = −0.749 (−1.115 to −0.383), *p* < 0.001, and I^2^ = 25.862 (low heterogeneity, low certainty of evidence). (Figure 6).

#### 3.4.3. Hospital Length of Stay

Six studies reported hospital length of stay with vitamin-C-containing treatment regimens. Their use was not associated with a significant reduction in hospital stay; MD = 1.321 (−1.073 to 3.714), *p* = 0.279, and I^2^ = 20.95 (low heterogeneity, very low certainty of evidence).

#### 3.4.4. ICU Length of Stay

Nine studies reported ICU length of stay with vitamin-C-containing treatment regimens. Their use was not associated with a significant decrease in ICU length of stay; MD = −0.804 days (−2.374 to 0.766), *p* = 0.315, I^2^ = 52.347 (moderate heterogeneity, very low certainty of evidence).

#### 3.4.5. Duration of Vasopressors Use

Twelve studies reported the duration of vasopressor support in patients with vitamin C therapy. Their use was associated with statistically significant reduced time on vasopressors; MD = −1.034 (−1.622 to −0.445), *p* = 0.001, I^2^ = 88.966 (high heterogeneity, very low certainty of evidence).

### 3.5. Subgroup Analysis

#### 3.5.1. Monotherapy

Treatment regimens with vitamin C monotherapy were associated with a statistically significant reduction in mortality: OR = 0.515 (0.390 to 0.680), *p* < 0.001, and I^2^ = 0 (low heterogeneity, moderate certainty of evidence); a reduction in ICU length of stay: MD = −2.551 days (−4.577 to −0.525), *p* = 0.014, and I^2^ = 37.983 (low heterogeneity); and a reduced time on vasopressors: MD = −1.180 days (−2.021 to −0.240), *p* = 0.006, and I^2^ = 92.664 (high heterogeneity). No significant change in the length of hospital stay or SOFA scores was reported.

#### 3.5.2. Combination Therapy

In some trials, vitamin C was used as a combination therapy with hydrocortisone and thiamine as an intravenous HAT regimen or enteral feed formula containing vitamin C. These combination regimens were associated with a statistically significant reduction in SOFA score: MD = −0.690 (−1.056 to −0.324), *p* < 0.001, and I^2^ = 0 (low heterogeneity); and reduction in duration of vasopressor use: MD = −0.933 days (−1.341 to −0.525), *p* < 0.001, and I^2^ = 17.709 (low heterogeneity). There was no statistical difference observed in mortality, ICU, or hospital length of stay.

### 3.6. Sensitivity Analysis

A sensitivity analysis for mortality was performed with the removal of studies identified with a possible high risk of bias, and no significant change in the results was noted: OR = 0.696 (0.538 to 0.900), *p* = 0.006 [23,26,29,34]. Similarly, studies with imputed standard deviations were excluded from SOFA score studies, but no significant change in the results was noted: MD = −1.062 (−1.458 to −0.666), *p* < 0.001 [13,24,29].

### 3.7. Summary of Results

Overall, treatment regimens containing vitamin C were associated with a statistically significant reduction in mortality, SOFA scores, and duration of vasopressor requirement compared to the control group. No statistically significant difference was found in ICU or hospital length of stay. A summary of the primary outcome (mortality) with the subgroup analysis results is shown in Table 2. A summary of the secondary outcomes is shown in Table 3.

## 4. Discussion

This meta-analysis included only 23 randomized control trials, while retrospective studies were excluded from the analysis. In 20 of these studies, the studied group received intravenous treatment regimens containing vitamin C, while the other 3 studies included study groups that received enteral regimens containing vitamin C. Interventions in some of these selected studies included vitamin C alone (monotherapy group) and the standard of care. In other studies, vitamin C was combined with other treatments, including thiamine and hydrocortisone (combination therapy group). Interestingly, our results revealed that patients treated with the vitamin C had significantly improved outcomes across various measures compared with the standard of care, most importantly mortality: OR = 0.778 (0.635 to 0.954), *p* = 0.016. The exact physiological mechanism by which vitamin C exerts these effects is still not fully understood.

The role of free radicals and reactive oxygen species (ROS) has long been a subject of interest in explaining physiology and disease pathology. Halliwell et al. explored the role of free radicals and ROS in many medical arenas, including cancers and neurodegenerative diseases [35]. Ascorbic acid provides an electron to another molecule, after which it transforms into an ascorbyl radical, which is the oxidized form of ascorbic acid and a relatively stable free radical [36]. Ascorbic acid in sepsis has specifically gained more scientific attention in the past years. Outcomes including 28-day mortality, ICU and hospital length of stay, vasopressor requirements, changes in the SOFA score, and others were studied in various articles [22,26,36].

Ascorbic acid in septic patients reportedly decreases inflammatory markers (c-reactive protein and procalcitonin) [16]. Vitamin C possibly inhibits the TNF-a-induced phosphorylation of inhibitory kappa-B kinase (IkB kinase), suppressing NF-kB and histamine levels in the blood [37,38]. It may also suppress high mobility group box-1 (HMGB-1) release from the LPS-activated macrophages, leading to improved survival and reduced (HMGB-1) levels in the septic host [3,37,38,39].

High concentrations of vitamin C in macrophages, leukocytes, and lymphocytes enhance their phagocytic potential, oxidative killing, and promoting lymphocyte proliferation. The neutrophil extracellular trap (NET) is a novel mechanism deployed by the host cells to kill pathogens. Nevertheless, a disproportionate NET during sepsis can harm host organs, and vitamin C is shown to alter and mediate the NET formation during sepsis [40,41].

Studies have shown that adding vitamin C into a medium containing lymphocytes from the peripheral circulation reduces the lipopolysaccharide (LPS)-induced production of the proinflammatory cytokines, specifically TNF-α and IFN-γ [42], and increases the production of the anti-inflammatory cytokine IL-1 [42]. Similarly, other studies conducted in vitro show that mixing ascorbic acid with monocytes isolated from the peripheral circulation of patients with underlying pneumonia resulted in the diminished production of the proinflammatory cytokines TNF-α and IL-6 [43] and the augmentation of IFN levels [44,45,46]. Therefore, there is evidence that ascorbic acid influences cytokine regulation, including the modulation in the cytokine levels deranged in sepsis.

Several meta-analyses have analyzed the role of vitamin C in multiple diseases [47,48]; some studies have focused on critically ill patients [49,50], including heterogeneous diseases and patient populations in the analysis. In contrast, others have analyzed the role of vitamin C in specific patient populations, such as COVID-19 and respiratory tract infections [51,52]. Several studies have addressed the role of hydrocortisone, ascorbic acid, and thiamine in sepsis [53,54,55,56,57], while others have considered vitamin-C-containing regimens [27,58,59]. Some previous studies, which included all regimens containing vitamin C, have indicated a trend toward lower short-term mortality [47,58]. Studies including combination therapies have yielded heterogeneous results ranging from no difference in mortality [57] to significant changes in mortality [55]. Different inclusion parameters and study populations partially explain the heterogeneity of outcomes in different reviews. Based on conflicting data from multiple trials, “Surviving sepsis guidelines” recommended against routine use of intravenous vitamin C based on 7 RCTs included in their analysis. More studies with increasing data have been published since the publication of the guidelines.

Most of the previous meta-analyses have reported an improvement in SOFA scores with vitamin C use [54,57,60]. Since their publication, other trials have been performed. Our analysis adds to the growing evidence of the positive impact of vitamin C use in the septic patient population. Our study’s major strength is that it only included randomized trials and excluded all observational studies in order to generate the highest strength of evidence possible. We included all the latest literature on the topic to the best of our efforts. Our results demonstrating a reduction in mortality and SOFA score improvement align with most previous studies on the topic. In addition to the amelioration observed in the clinical status and significant improvement in patient care, this remains an important finding because intravenous vitamin C is readily available, very affordable, and has very limited secondary side effects.

This study, as a meta-analysis, remains a retrospective review and generates various biases. Heterogeneity remains a significant challenge, given the inclusion of studies with different patient populations in different stages of diseases. Secondly, there is no universally accepted dose of vitamin C, which could be defined as a “high dose,” and little to no data exist to guide frequency, dosing, and route of administration. Some of the studies utilized an enteral route of drug administration, which in the septic population suffers from erratic absorption and significant drug–drug interactions. Most studies did not report complete patient data, age groups, and reasons for mortality, which also weakens the overall certainty of the evidence. Many studies reported medians for reporting their outcomes, which showed significant deviation from the normal distribution and had to be excluded from the analysis, thereby decreasing the population pool [13,14,28,29,31]. Furthermore, the combination of intervention drugs with other drugs, differences in ICU protocols, and hospital policies also contribute to the high heterogeneity and low certainty of the outcomes.

Overall, we made our greatest attempt to include all relevant published studies and extract data for analysis. There are several ongoing trials on this topic, and soon, we might have more definite answers regarding the efficacy, dosing, route, and timing of the administration of vitamin C in the septic patient population [61,62,63].

### Clinical Implications and Future Directions

Based on this pooled evidence, we advocate using vitamin C as an adjunct to the standard of care for sepsis management, given the postulated reduction in mortality, low cost, and relatively safe adverse effect profile. There remains a need for a large, multi-center, randomized control trial with enough power to better understand and answer questions regarding the utility, timing of administration, route of delivery, and dosage of vitamin C in sepsis.

## Figures and Tables

**Table 1:** Baseline characteristics of included studies.

Study Name	No. of Patients	Type of Therapy	Inclusion Criteria	Exclusion Criteria	Primary Outcome	Secondary Outcome
Rosengrave, 2022 [12]	40	Monotherapy	Receiving intravenous antimicrobial therapy specifically for infection, receiving ≥ 5 μg/min noradrenaline or adrenaline, SOFA score ≥ 2	Age < 18 years, consent could not be obtained, patient not expected to survive 24 h, known G6PD deficiency, known or suspected, pregnancy or breastfeeding.	Vasopressor requirement	Vasopressor dose delivered, SOFA scores, oxygenation parameters, and length of ICU and hospital stay, and ICU and hospital mortality
Wacker, 2022 [13]	124	Monotherapy	Adult patients within 24 h of onset of septic shock	Inability to obtain written consent and initiate study drug within 24 h of eligibility, known history of nephrolithiasis, and shock occurring immediately following cardiac arrest.	28-day mortality	ICU mortality, time to lactate clearance, need for renal replacement therapy (RRT), changes in severity-of-disease index scores, and duration of ICU and hospital stay
Lv, 2021 [14]	117	Monotherapy	Admitted to the ICU, age 18–75 years, meet criteria of sepsis, no vitamin C treatment before admission	Terminal-stage patients, pregnant or lactating females, long-term use of hormones or immunosuppressive agents, active malignancy, mental disorders, autoimmune diseases	28-day mortality	Changes in the SOFA scores in the first 72 h after ICU admission, application time of vasoactive drugs, duration of time in ICU, procalcitonin clearance
Mahmoodpoor, Jun 2021 [15]	80	Monotherapy	Patients with severe pneumonia	Age 18–80 years, renal insufficiency, history of vitamin C usage during past 48 h., pregnancy or breastfeeding, life expectancy of fewer than 24 h, G6PD deficiency, DKA, nephrolithiasis	Days on mechanical ventilation, vasopressor use and dose, 28-day mortality	CRP, procalcitonin, ICU length of stay (LOS)
Fowler III, 2014 phase I [16]	24	Monotherapy	Presence of a SIRS, suspected or proven infection, presence of sepsis-induced organ dysfunction	Terminal Cancer or Expected Survival < 24 h	Ascorbic acid safety and tolerability	Days on vasopressor, ventilator-free days, ICU length of stay, 28-day mortality
Fowler III, 2019 [17]	167	Monotherapy	Should meet 2 of 4 SIRS criteria with the following: Mechanically ventilation, PaO2:FiO2 < 300 mm Hg, bilateral opacities by chest radiography within 1 week of known clinical insult, new or worsening respiratory symptoms without evidence of left atrial hypertension, suspected or proven infection	Age < 18 years, non-English speaking, >48 h had elapsed since they met ARDS criteria, pregnant or breastfeeding, not expected to survive 24 h, receiving home oxygen greater than 2 L/min: history of interstitial lung disease, DAH, DKA, nephrolithiasis	Change in SOFA score from baseline to 96 h, change in plasma biomarkers of inflammation (CRP) and vascular injury (thrombomodulin levels)	All-cause 28-day mortality, ventilator-free days, ICU-free days, hospital-free days, SOFA score, vasopressor use, GCS, creatinine level
Zabet MH, 2016 [18]	28	Monotherapy	Patients with septic shock as per Surviving Sepsis Campaign and with the following criteria: Presence of SIRS with suspected or proven infection and presence of sepsis-induced organ dysfunction	Concomitant use of other antioxidants, corticosteroids administration, contraindication for high-dose ascorbic acid including bilateral ureteric obstruction, chronic hemodialysis, iron overload, oxalate stone formers, hemochromatosis, and glucose-6-phosphate dehydrogenase (G6PD) deficiency	Vasopressor dose and duration	Duration of Intensive Care Unit (ICU) stay and 28-day mortality
Nabil Habib, 2017 [19]	100	Monotherapy	Admitted with the diagnosis of Septic shock	Pregnant and lactating females, history of oxalate nephrolithiasis, G6PD deficiency, PNH, hereditary hemochromatosis, shock not due to sepsis, or patients with mixed type of shock	Need for organ supportive measures and Length of ICU stay	In-ICU mortality
Ferron-Celma, 2009 [20]	20	Monotherapy	Age > 18, meets sepsis criteria, undergoing abdominal surgery, POSSUM score > 30%	Age < 18 years old, unable to obtain consent	Neutrophil apoptosis via Fas receptor expression and caspase-3, poly (ADP-ribose) polymerase (PARP), and Bcl-2 levels in neutrophils	Mortality
Galley, 1997 [21]	30	Combination therapy	Meet septic shock criteria, receiving vasoconstrictor therapy (epinephrine, norepinephrine, dopamine)	Age < 21 years old, unable to obtain consent	Total lipid peroxidase, malondialdehyde, Arginine: Citrulline, total Nitrite, reactive redox iron, total antioxidant capacity	Mortality
Hussein, 2021 [22]	94	Combination therapy	With septic shock on ICU admission or development during ICU stay	Pregnancy and lactation, contraindication to any of the components of the triple therapy regimen, G6PD deficiency, receiving another type of steroid during their stay, immunosuppressive medications, cancer patients, DNR/DNI patients	28-day in-hospital mortality and ICU mortality	Shock time and duration on pressors
Wani, 2020 [23]	100	Combination therapy	Admitted with a diagnosis of sepsis and septic shock with a serum lactate level of >2 mmol/L	Age < 18 years, Pregnancy	In-hospital mortality	30-day mortality, hospital LOS, duration of vasopressor therapy, lactate clearance, change in serum lactate, and the SOFA score over the first 4 days
Sevransky, 2021 [24]	501	Combination therapy	Suspected or confirmed infection, ICU admission, acute respiratory and/or cardiovascular organ dysfunction attributed to sepsis and requiring vasopressor (regardless of agent or dose)	Age < 18 years old, cardiovascular or respiratory organ failure requiring treatment, life expectancy < 30 days, DNR/DNI and current hospitalization > 30 days at the time of randomization, allergies to intervention, primary hyperoxaluria, oxalate nephropathy, G6PD deficiency, pregnancy, or known active breastfeeding, incarceration	Number of ventilator and vasopressor-free days in the first 30 days following the day of randomization	30-day mortality
Moskowitz, 2020 [25]	205	Combination therapy	Age > 18 years, with suspected or confirmed infection and were receiving a vasopressor because of sepsis	Allergic to study drug components had a clinical indication for any of the study drugs, symptomatic kidney stones within the last year, G6PD deficiency, hemochromatosis, ESRD, or RRT, not expected to survive 24 h	Change in the (SOFA) score between enrollment and 72 h	Renal failure and 30-day mortality
Mohamed, 2020 [26]	88	Combination therapy	Adult non-pregnant patients with septic shock with initiation inotropic/presser support within 6 h of presentation	Patients with burns, limitations of care due to terminal illness, acute liver failure, pregnancy	Mortality during the inpatient stay	Time to shock reversal, change in SOFA score over 72 h, need for mechanical ventilation, incidence of new-onset AKI and ICU/hospital LOS.
Iglesias, 2020 [27]	137	Combination therapy	Adults > 18 years of age with a primary diagnosis of sepsis or septic shock or diagnosis of sepsis or septic shock within 12 h of admission to the ICU	Age < 18, pregnancy, DNR/DNI, terminal end-stage disease, required immediate surgery, HIV positive with a CD4 < 50 mm^2^, G6PD deficiency, transferred from another hospital	Resolution of shock and change in SOFA score.	28-day mortality, ICU mortality, hospital mortality, procalcitonin clearance, hospital LOS, ICU LOS, ventilator-free days.
Hwang, 2020 [28]	111	Combination therapy	Adult patients (19–89 years old) presented and were diagnosed with septic shock.	Transferred from another hospital with vasopressor administration or mechanical ventilator support, limitations to treatment due to terminal disease or DNR/DNI, receiving vitamin C or thiamine before enrolment, cardiac arrest before enrolment, renal or ureteral stones	The change in SOFA score between the time of admission and 72 h after admission	Shock reversal and 28-day mortality
Fujii, 2020 [29]	216	Combination therapy	Diagnosed with septic shock within a maximum of 24 h before enrollment, vasopressor dependent for at least 2 h at the time of enrollment	Age < 18 years, DNR/DNI, imminent death, diagnosis of septic shock longer than 24 h ago, known or suspected disease with a strong indication or contraindication for any of the study drugs, another indication for hydrocortisone use other than septic shock.	Time alive and free of vasopressors at day 7 after randomization	28-day, 90-day, ICU, and hospital mortality, 28-day cumulative vasopressor free days, 28-day cumulative mechanical ventilation-free days, 28-day renal replacement therapy–free days, change in SOFA score at day 72 h, 28-day ICU-free days and hospital LOS
Chang P, 2020 [30]	80	Combination therapy	Meeting the diagnostic criteria for sepsis, >18 years of age and procalcitonin > 2 ng/mL when entering the ICU	Pregnancy, terminal disease or DNR/DNI orders, major bleeding, cardiogenic shock, paraquat poisoning, persistent nonremovable infection sources.	28-day all-cause mortality	Organ protection, procalcitonin reduction, adverse events related to therapy
Niu, 2019 [31]	234	Monotherapy	Age 18–75, meet diagnostic criteria for sepsis, no vitamin C used earlier in the hospitalization	Terminal-stage patients, pregnant or lactating females, long-term use of hormones or immunosuppressive agents, active malignancy, mental disorders	28 Day mortality	72 h change in SOFA, duration of vasopressors, elimination of Calcitonin, ICU length of stay
Beale, 2008 [32]	55	Enteral pharma-co-nutrition supplement (Intestamin)	Age > 18 years old, meeting SIRS criteria and at least one organ dysfunction occurring within 24 h. of ICU admission, APACHE II score >10, expected LOS in the ICU > 3 days, and indication for enteral nutrition for > 5 days	Cardiogenic shock or severe congestive heart failure NYHA class IV), severe, Pre-existing, parenchymal liver disease with clinically significant portal hypertension (Childs C), COPD, chronic renal failure requiring dialysis, pregnancy, AIDS, Immunosuppression, active cancer treatment	SOFA score	Mortality, length of stay and organ dysfunction
Pontes-aruda, 2006 [33]	103	Continuously tube-fed with a diet enriched with Eicosapentaenoic acid, Gamma Linolenate, and antioxidants	Age > 18 years old with clinical diagnosis of severe sepsis or septic shock, requiring mechanical ventilation and enteral access.	Age < 18 years, significant limitation of survival prognosis and/or incurable disease, chronic renal insufficiency, acute pancreatitis without established origin, head trauma with a GCS score < 5, recent stroke or subarachnoid hemorrhage, immunologic suppression, no indication for enteral nutrition or imminence of receiving parenteral nutrition, receiving partial parenteral nutrition, presence of uncontrolled diarrhea, recent GIB, planned to wean from mechanical ventilation	28-day mortality	Changes in oxygenation status, time receiving mechanical ventilation, period in the intensive care setting, and development of new organ dysfunctions.
Schneider 2011 [34]	58	Enteral supplement Intestamin	Age 18–75 years, sepsis, or SIRS (APACHEscore 10–30), absence of severe gastrointestinal tract or metabolic diseases, enteral feeding within 48 h of admission with obtained written consent.	Expected patient survival less than 6 days, pregnancy or lactation, hemodynamically unstable, severe liver disease with cytolysis, gastrointestinal surgery in the last 4 weeks, severe enteritis/colitis, short intestine syndrome, GIB requiring intervention, and inability to enterally feed	ICU LOS	Hospital LOS, mortality, ventilator requirement and vasopressor requirement.

**Table 2:** Summary of results for the primary outcome (Mortality).

Outcomes	Relative Risk (95% CI)	Risk Difference (95% CI)		No. of Studies (Total Patients)	Certainty (GRADE)
		Risk with Control	Risk Difference with Vitamin C		
Overall Mortality	OR = 0.778 (0.635 to 0.954)	32 deaths per 100	6 less deaths per 100 (1 fewer to 10 fewer)	23 (2705)	Low ^a^
Mortality with IV Vitamin C regimens	OR = 0.780 (628 to 0.968)	37 deaths per 100	6 less deaths per 100 (1 fewer to 10 fewer)	20 (2489)	Low ^b^
Mortality with Enteral Vitamin C regimens	OR = 0.782 (0.367 to 1.665)	37 deaths per 100	5 less deaths per 100 (21 fewer to 10 more)	3 (216)	Very Low ^c^
Mortality with Vitamin C Monotherapy	OR = 0.515 (0.390 to 0.680)	42 deaths per 100	15 fewer deaths per 100 (21 fewer to 9 fewer)	10 (941)	Moderate ^d^
Mortality with Combination regimens containing Vitamin C	OR = 1.023 (0.836 to 1.251)	34 deaths per 100	No significant difference (4 fewer to 5 more)	13 (1764)	Low ^e^

^a^ Downgraded two levels for limitation in design (multiple routes of administration, doses, timing) and risk of bias. ^b^ Downgraded two levels for design limitations (variable dosing and timing) and risk of bias. ^c^ Downgraded three levels for imprecision (wide confidence intervals) and limitations in design (enteral feeding with variable dosing and absorption). ^d^ Downgraded one level for design limitations (variable dosing and timing) ^e^ Downgraded two levels for severe limitations in design (multiple concurrent interventions).

**Table 3:** Summary of results (secondary outcomes).

Outcomes	Number of Studies	Sample Size		Absolute Effect Size (95% CI)	Certainty (GRADE)
		Control	Vitamin C		
SOFA Score	11	819	829	0.75 lower (−1.12 lower to −0.38 lower)	Low ^a^
Duration of vasopressor use	9	449	449	1.03 fewer days (1.62 fewer to 0.45 fewer)	Very Low ^b^
Duration of vasopressor use	9	449	449	1.03 fewer days (1.62 fewer to 0.45 fewer)	Very Low ^b^
ICU length of stay	12	333	333	0.81 fewer days (1.64 fewer to 0.02 more)	Very Low ^c^
Hospital length of stay	6	239	239	1.32 more days (1.07 fewer to 3.71 more)	Very Low ^d^

^a^ Downgraded two levels for limitation in design (multiple routes of administration, doses, timing) and risk of bias. ^b^ Downgraded three levels for inconsistency, limitation in design (multiple routes of administration, doses, timing), and risk of bias. ^c^ Downgraded three levels for risk of bias, imprecision (wide confidence intervals), and limitations in design (variable dosing and timing). ^d^ Downgraded three levels for risk of bias, imprecision (wide confidence intervals), and limitations in design (variable hospital protocols, drug dosing, and timing).

## Data Availability

Not applicable.
